# Compensatory Processes in Striatal Neurons Expressing the Tyrosine Hydroxylase Gene in Transgenic Mice in a Model of Parkinson’s Disease

**DOI:** 10.3390/ijms242216245

**Published:** 2023-11-13

**Authors:** Dmitry Troshev, Alyona Bannikova, Victor Blokhin, Ekaterina Pavlova, Anna Kolacheva, Michael Ugrumov

**Affiliations:** Laboratory of Neural and Neuroendocrine Regulations, Koltzov Institute of Developmental Biology of the Russian Academy of Sciences, 119334 Moscow, Russia; dmitry.vad.troshev@gmail.com (D.T.); alyona8annikova@yandex.ru (A.B.); victor.blokhin@hotmail.com (V.B.); guchia@gmail.com (E.P.); annakolacheva@gmail.com (A.K.)

**Keywords:** dorsal striatum, dopamine, tyrosine hydroxylase, aromatic L-amino acid decarboxylase, 1-methyl-4-phenyl-1,2,3,6-tetrahydropyridine, Parkinson’s disease, transgenic mice

## Abstract

The mammalian striatum is known to contain non-dopaminergic neurons that express dopamine (DA)-synthesizing enzymes and produce DA, responsible for the regulation of motor function. This study assessed the expression of DA-synthesizing enzymes in striatal neurons and their role in DA synthesis in transgenic mice expressing the green fluorescent protein (GFP) gene under the tyrosine hydroxylase (TH) gene promoter in a 1-methyl-4-phenyl-1,2,3,6-tetrahydropyridine (MPTP) model of Parkinson’s disease (PD). We showed that, in Parkinsonian animals, the number of neurons expressing the TH gene increased by 1.9 times compared with the control (0.9% NaCl), which indicates a compensatory response to the DAergic denervation of the striatum. This assumption is supported by a 2.5-fold increase in the expression of genes for TH and transcription factor Nurr1 and a 1.45-fold increase in the expression of the large amino acid transporter 1 gene. It is noteworthy that, in Parkinsonian mice, in contrast to the controls, DA-synthesizing enzymes were found not only in nerve fibers but also in neuronal cell bodies. Indeed, TH or TH and aromatic L-amino acid decarboxylase (AADC) were detected in GFP-positive neurons, and AADC was detected in GFP-negative neurons. These neurons were shown to synthesize DA, and this synthesis is compensatorily increased in Parkinsonian mice. The above data open the prospect of improving the treatment of PD by maintaining DA homeostasis in the striatum.

## 1. Introduction

The key link in the regulation of motor function in animals and humans is dopamine (DA) neurotransmission in the striatum, and its failure as a result of DAergic deafferentation leads to the development of Parkinson’s disease (PD) [1,2,3]. PD is the second most socially significant neurodegenerative disease after Alzheimer’s disease in terms of severity, incidence, and cost, with global prevalence of PD increasing by 74.3% between 1990 and 2016 [4]. The current symptomatic treatment of PD with DA agonists and L-3,4-dihydroxyphenylalanine (L-DOPA), the immediate precursor of DA, becomes ineffective and even leads to dyskinesia over time [5,6,7], making its improvement necessary.

One of the promising approaches to improving pharmacotherapy for PD may be based on the study of compensatory processes aimed at maintaining DA homeostasis in the striatum and the development of pharmacological control of these processes. In this regard, the study of DA synthesis with non-DAergic neurons in the striatum is particularly promising. Indeed, many years ago, neurons containing enzymes for DA synthesis were immunohistochemically discovered in the striatum [8,9,10,11,12,13,14]. The number of such neurons varies from tens in rodents to hundreds in primates, including humans [9,11,12,13,15]. Of particular interest is the fact that, with DAergic deafferentation of the striatum, the number of striatal neurons expressing DA-synthesizing enzymes increases significantly, which is the case for patients with PD and neurotoxic animal models of PD [16,17,18,19,20].

Significant progress in the study of striatal neurons expressing DA-synthesizing enzymes has been made because of the use of transgenic mice expressing the green fluorescent protein (GFP) gene under the promoter of the tyrosine hydroxylase (TH) gene, the first rate-limiting enzyme of DA synthesis. It has been shown that the striatum of these intact mice contain about three thousand TH-expressing neurons [21]. In turn, immunohistochemistry has revealed three populations of non-DAergic neurons containing only one or both enzymes of DA synthesis but lacking the DA transporter [14,15,21,22]. Notably, in transgenic mice, as in wild-type mice, neurons expressing only TH, as well as any other neurons expressing aromatic L-amino acid decarboxylase (AADC) but lacking TH, synthesize DA in cooperation [21,23].

Based on the above, the aim of this work was to study compensatory changes in the non-DAergic neurons of the striatum in transgenic mice expressing the GFP gene under the TH gene promoter, with an emphasis on the participation of these neurons in maintaining DA homeostasis in the neurotoxic 1-methyl-4-phenyl-1,2,3,6-tetrahydropyridine (MPTP) model of PD.

## 2. Results

### 2.1. Estimation of the Number of Striatal Neurons Expressing the Tyrosine Hydroxylase Gene in Mice When Modeling Parkinson’s Disease and in the Control

**Figure 1:**
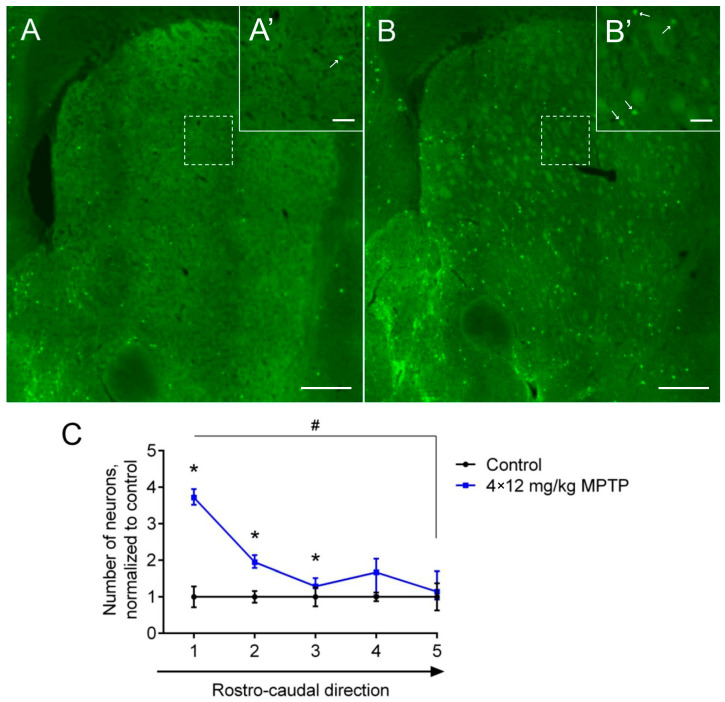
Neurons expressing the tyrosine hydroxylase (TH) gene and containing green fluorescent protein (GFP) in the striatum of transgenic mice in the control (0.9% NaCl) and two weeks after four injections of 1-methyl-4-phenyl-1,2,3,6-tetrahydropyridine (MPTP) at a single dose of 12 mg/kg. (**A**,**A’**) Microphoto of TH neurons in mice of the control group at low (**A**) and high (**A’**) magnification. (**B,B’**) Microphoto of TH neurons in mice of the experimental group at low (**B**) and high (**B’**) magnification. (**C**) Quantitative characteristics of the distribution of TH neurons in five segments of the dorsal striatum in the rostrocaudal direction (each segment is 320 μm thick) in mice two weeks after MPTP administration and in the control. The groups were compared for normality by using the Shapiro–Wilk test. Statistical analysis of unpaired values was performed using the Mann–Whitney test compared with the control group (* *p* ≤ 0.05). Statistical analysis of paired values was performed using the Friedman test and Dunn’s multiple comparisons test (# *p* ≤ 0.05). Data are presented as medians and interquartile ranges. *n* = 4 for each group. Scale: 300 µm for (**A**,**B**); 50 µm for (**A’**,**B’**). Arrows, GFP-containing neurons.

For the ease of counting neurons, the striatum was divided into five equal segments in the rostrocaudal direction, each 320 µm thick. In these segments of the striatum in the control mice, the total number of GFP-containing neurons expressing the TH gene was 1697. The number of such neurons increased by 91.59% after the DAergic denervation of the striatum in MPTP-treated mice (*p* = 0.0079) (Figure 1A,A’,B,B’). At the same time, an increase in the number of GFP-containing neurons was observed in three segments of the striatum out of the five segments analyzed (*p* = 0.0286) (Figure 1C). The increase in the number of neurons expressing the TH gene was especially pronounced in animals of the experimental group, predominantly in the first segment of the striatum (*p* = 0.0176) (Figure 1C).

### 2.2. Colocalization of Green Fluorescent Protein, Tyrosine Hydroxylase, and Aromatic L-amino Acid Decarboxylase

**Figure 2:**
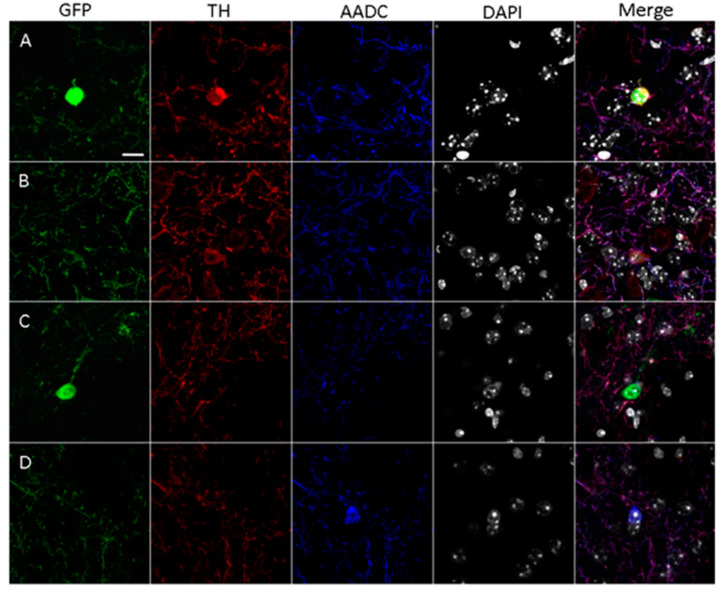
Confocal microscopy of the striatum of transgenic mice two weeks after four injections of 1-methyl-4-phenyl-1,2,3,6-tetrahydropyridine at a single dose of 12 mg/kg. (**A**) Neuron cell body containing green fluorescent protein (GFP, green), immunopositive for tyrosine hydroxylase (TH, red) and for aromatic L-amino acid decarboxylase (AADC, blue), with the nucleus stained with 4′,6-diamidino-2-phenylindole (DAPI, gray). (**B**) Neuron cell body containing GFP, immunopositive for TH but immunonegative for AADC. (**C**) Neuron cell body containing GFP, immunonegative for TH and for AADC. (**D**) Neuron cell body lacking GFP, immunonegative for TH but immunopositive for AADC. Scale: 10 µm.

After double immunostaining for TH and AADC, only GFP-containing neurons lacking TH-immunopositive and AADC-immunopositive materials were found in the striatum of the control mice. In mice of the experimental group, in contrast to mice in the control group, we found single neurons (from 1 to 5 neurons per section) that were stained with GFP and immunopositive only for TH or for both TH and AADC (Figure 2A,B). However, most striatal neurons containing GFP and, hence, expressing the TH gene were immunonegative for TH and AADC (Figure 2C). In addition, single AADC-immunopositive neurons (1–2 per section) unstained with GFP were discovered (Figure 2D).

The distribution of these neurons shows certain zonation (Figure S1). Neurons containing only GFP are located mainly along the lateral ventricles of the brain, as well as in the central region of the dorsal striatum and along its ventral border. Neurons containing GFP and immunostained for TH and AADC are located predominantly in the central part of the dorsal striatum and along its ventral edge. Neurons containing GFP and immunostained for TH, as well as neurons immunostained for AADC but lacking GFP, are randomly distributed throughout the striatum (Figure S1).

### 2.3. Assessment of Cooperative Dopamine Synthesis in the Striatum of Transgenic Mice When Modeling Parkinson’s Disease

**Figure 3:**
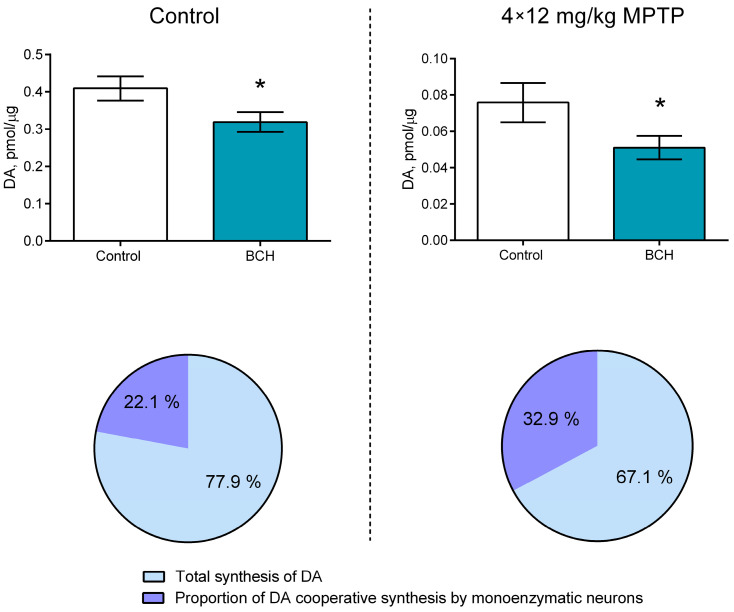
Comparison of the contribution of cooperative dopamine (DA) synthesis to total DA synthesis in the striatum of mice two weeks after four subcutaneous injections of 1-methyl-4-phenyl-1,2,3,6-tetrahydropyridine (MPTP) at a single dose of 12 mg/kg and in the striatum of control mice after subcutaneous injection of saline. The groups were compared for normality by using the Shapiro–Wilk test. Statistical analysis was performed using the paired *t*-test (* *p* ≤ 0.05). The data are presented as mean ± SEM. *n* = 9 for each group. BCH, 2-aminobicyclo [2.2.1]heptane-2-carboxylic acid.

The administration of MPTP to the animals of the experimental group resulted in a decrease in the total DA content in the striatum sections and in the incubation medium by 81.5% compared with the animals of the control group (Figure 3). The total DA content after the incubation of sections with 2-aminobicyclo [2.2.1]heptane-2-carboxylic acid (BCH) was 22.1% lower than after incubation without BCH in the control group animals (*p* = 0.0448; Figure 3). At the same time, the total DA content after the incubation of sections with BCH was 32.9% lower than after incubation without BCH in the animals of the experimental group (*p* = 0.0073) (Figure 3). Thus, the relative contribution of cooperative DA synthesis to total DA synthesis in the striatum increased by 48.9% after the MPTP-induced DAergic denervation of the striatum (*p* = 0.0314).

### 2.4. Gene Expression of Functionally Significant Proteins in Striatal Neurons Expressing the Tyrosine Hydroxylase Gene in the Control and When Modeling Parkinson’s Disease

#### 2.4.1. Isolation of Striatal Neurons Expressing the Tyrosine Hydroxylase Gene

**Figure 4:**
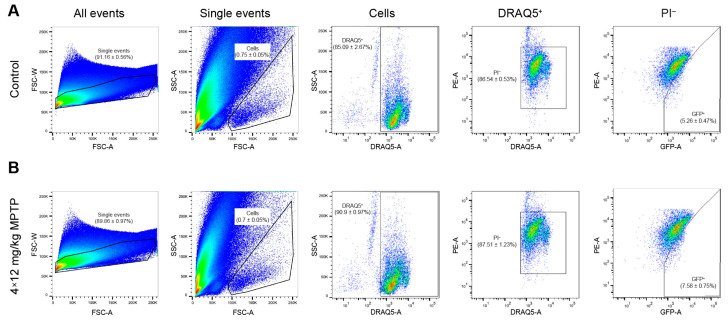
Isolation of green fluorescent protein (GFP)-containing striatal neurons with nuclei stained with DRAQ5. (**A**) Isolation of neurons from the striatum of control mice after subcutaneous injections of saline. (**B**) Isolation of neurons from the striatum of experimental mice after four subcutaneous injections of 1-methyl-4-phenyl-1,2,3,6-tetrahydropyridine (MPTP) at a single dose of 12 mg/kg. The histograms are presented as the density plots. For each cell suspension, at least 1,000,000 events were analyzed. For each histogram, *n* = 8 samples (two mice per sample). The data are presented as mean ± SEM. FSC, forward scatter; PE, phycoerythrin (detection channel); PI, propidium iodide; SSC, side scatter.

While obtaining striatal neurons containing GFP and, therefore, expressing the TH gene, we used the same gating strategies that we used to isolate neurons from the striatum of intact transgenic mice. In the transgenic animals of the experimental group, there were 44.1% more such neurons in the cell suspension than in the animals of the control group (*p* = 0.0223) (Figure 4A,B).

#### 2.4.2. Assessment of the Gene Expression of Functionally Significant Proteins in Striatal Neurons Expressing the Tyrosine Hydroxylase Gene in the Control and When Modeling Parkinson’s Disease

**Figure 5:**
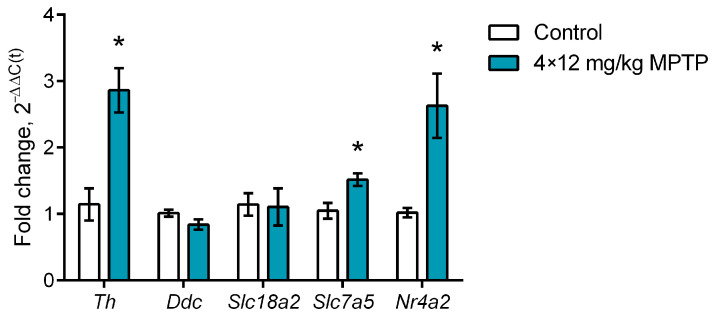
Expression of *Th* (tyrosine hydroxylase gene), *Ddc* (aromatic L-amino acid decarboxylase gene), *Slc18a2* (vesicular monoamine transporter 2 gene), *Slc7a5* (large amino acid transporter 1 gene), and *Nr4a2* (transcription factor Nurr1 gene) in sorted striatal neurons expressing the tyrosine hydroxylase gene in mice 2 weeks after four injections of 1-methyl-4-phenyl-1,2,3,6-tetrahydropyridine (MPTP) at a single dose of 12 mg/kg or saline in the control. The groups were compared for normality by using the Shapiro–Wilk test. Statistical analysis was performed using the unpaired *t*-test (* *p* ≤ 0.05 compared with the control group). The data are presented as mean ± SEM. *n* = 7 or 8 for each group.

When assessing the gene expression of functionally significant proteins in sorted striatal neurons expressing the TH gene, we showed that, when modeling PD, the gene expression of TH increased by 150.2% compared with the control (*p* = 0.0013), while the gene expression of the transcription factor Nurr1 increased by 158.4% compared with the control (*p* = 0.0053) (Figure 5). There was also a 44.9% increase in large amino acid transporter 1 (LAT1) gene expression compared with the control (*p* = 0.008) (Figure 5). The gene expression of AADC and vesicular monoamine transporter 2 (VMAT2) in striatal neurons expressing the TH gene did not change in animals treated with MPTP.

## 3. Discussion

In contrast to our previous work, which assessed the number of neurons expressing DA-synthesizing enzymes in the striatum of intact transgenic mice aged 8–12 weeks [21], in this study, we used older mice, aged 20–24 weeks. These animals are more suitable for modeling sporadic PD since, clinically, this disease manifests itself in older people. In fact, the total number of striatal neurons containing GFP and, therefore, expressing the TH gene in older mice was 1.8 times less than in younger mice [21]. It is important to note that, in this work, as in our previous study [21], at least a part of the striatal neurons contained GFP and, therefore, expressed the TH gene, as well as the AADC and VMAT2 genes. This fact indicates that this population of striatal neurons has a full set of chemical machinery that ensures the synthesis of DA and its storage in vesicles. However, it should be borne in mind that the VMAT2 protein cannot be detected with immunohistochemistry in neurons of the striatum [14,24], and therefore, the question of the mechanism of DA storage in vesicles and its release via exocytosis remains open and requires further development.

Surprisingly, in neurons expressing the TH and AADC genes in transgenic mice and in normal wild-type mice (control), the TH and AADC proteins encoded by these genes are not detected immunohistochemically in the cell bodies. However, both proteins are detected by this method in the processes (nerve fibers) of striatal neurons [21,23]. This can be explained either by the fact that TH and AADC are transported into neuronal processes immediately after synthesis or by the fact that these proteins change their conformation during transport, becoming immunoreactive only in neuronal processes [23].

The present study is focused on assessing changes in the number of non-DAergic neurons expressing DA-synthesizing enzymes and the DA synthesis caused by these neurons in the striatum of transgenic mice in a neurotoxic model of PD. To model PD, transgenic mice were subcutaneously administered MPTP four times at a single dose of 12 mg/kg with an interval of 2 h between injections, as was carried out in our previous study on wild mice when modeling PD at the early clinical stage [25].

According to our morphological data, the total number of neurons containing GFP and, therefore, expressing the TH gene increased by 1.9 times in the striatum of transgenic mice after its DAergic denervation using MPTP. According to the literature, the number of neurons expressing TH increases when modeling PD because of changes in the phenotype of existing striatal neurons and not because of neuronogenesis [13,22]. It is noteworthy that we observed the greatest increase in the number of these neurons, almost four times, in the most rostral segment of the rostrodorsal striatum, which receives the greatest DAergic innervation from neurons of the substantia nigra, which plays a key role in the regulation of motor behavior [26]. This raises the following question: what is the mechanism that drives TH gene expression in non-DAergic neurons in this area of the striatum in animal models of PD and in PD patients? In this regard, it is of particular interest that this region of the striatum loses the greatest number of axons of nigral DAergic neurons in animal models of PD [19,27]. Therefore, it is quite probable that the expression of the TH gene in striatal neurons—as with the expression of TH protein in other non-DAergic neurons, for example, in vasopressinergic neurons of the supraoptic nucleus and in neurons of the arcuate nucleus—is inhibited by catecholaminergic afferents through specific receptors [28,29]. This assumption should be tested in the future.

The increasing functional role of striatal neurons expressing the TH gene under PD modeling in transgenic mice is evidenced not only by an increase in the number of these neurons but also by a change in their functional activity. Indeed, it has been shown that, in GFP-containing neurons isolated by our original method from the striatum of transgenic mice [21], the expression of the TH and Nur1 genes increases approximately 2.5-fold in a model of PD compared with the control. It is known that Nur1 is a transcription factor that regulates the expression of the TH gene, as evidenced by its binding to the TH gene promoter [30,31] and localization in the nuclei of striatal neurons [32]. The increased expression of the TH gene that we noted in striatal neurons in the PD model is most probably the result of the increased expression of the Nurr1 gene and Nurr1 itself. From a physiological point of view, a simultaneous increase in the expression of the Nurr1 and TH genes in PD modeling can be considered one of the mechanisms of neuroplasticity, ensuring the maintenance of DA homeostasis in the striatum during its DAergic denervation.

In addition to the Nurr1 and TH genes, LAT1 gene expression increases by about 45% in striatal neurons in transgenic mice in a model of PD. This fact can also be considered a compensatory reaction aimed at maintaining DA homeostasis in the striatum by, in this case, increasing the efficiency of the uptake of large amino acids and L-DOPA, the metabolic precursors of DA, into striatal neurons. In contrast to the TH and LAT1 genes, the expression of the AADC and BMAT2 genes did not change in bienzymatic GFP-containing neurons in the striatum of transgenic mice when modeling PD, which suggests their different regulation.

From a physiological point of view, it was especially important to determine whether the cell bodies of striatal neurons contain only TH or TH and AADC proteins. This issue was resolved using immunohistochemistry, but only in the PD model. Indeed, only in the denervated striatum were we able to detect TH and AADC with immunohistochemistry in the cell bodies of neurons, and not only in their processes (nerve fibers), as in the control. These neurons contained (i) only TH in GFP-stained neurons, (ii) TH and AADC in GFP-stained neurons, and (iii) AADC in neurons not stained with GFP. These data are consistent with previous studies conducted on mice and monkeys [14,22], which showed that some striatal neurons contain both enzymes of DA synthesis. The detection of TH and AADC in the cell bodies of striatal neurons by using immunohistochemistry in transgenic mice when modeling PD and not in the control can be explained by the fact that, with the DAergic denervation of the striatum, either the rate of transport of TH and AADC from the neuron cell bodies to the processes decreases, or the synthesis of these proteins increases, which, in both cases, should lead to the accumulation of the DA-synthesizing enzymes in the cell bodies of neurons. Considering that even after the catecholaminergic deafferentation of the striatum, not all GFP-containing neurons immunostain for TH or for TH and AADC; it can be assumed that many more GFP-containing neurons synthesize these enzymes, but their content in cell bodies is below the resolution of immunohistochemistry.

Previous studies have shown that the final secretory product of monoenzymatic TH neurons is L-DOPA [33], which may act as a neurotransmitter or neuromodulator of catecholamine receptors [34,35,36,37]. In turn, the secretory product of monoenzymatic neurons containing AADC and of bienzymatic neurons containing TH and AADC is DA. In addition, we are the first to show while studying the hypothalamic arcuate nucleus that L-DOPA, which is secreted by monoenzymatic TH neurons, is captured by LAT1 in monoenzymatic neurons or any other neurons expressing AADC for DA synthesis [38]. Our further study has shown that this so-called cooperative synthesis of DA in the striatum is a compensatory reaction aimed at minimizing the DA deficit resulting from the DAergic deafferentation of these brain regions [23]. Proceeding from this idea, one of the objectives of the present study was to assess the cooperative synthesis of DA by monoenzymatic TH neurons and AADC-containing neurons in transgenic mice in normal conditions (control) and in the PD model. To solve this problem, we used a previously developed method based on an inhibition of LAT1 during the incubation of brain slices containing monoenzymatic TH neurons and any other neurons expressing AADC but lacking TH. The inhibition of LAT1 should prevent the uptake of L-DOPA secreted by monoenzymatic TH neurons into any AADC-containing neurons, resulting in decreased DA synthesis. In contrast to previous studies of the arcuate nucleus [38] and striatum [23], where large neutral amino acids were used as a LAT1 inhibitor, in this study, we used BCH, a synthetic non-metabolizable inhibitor with a higher affinity for this membrane transporter [39]. As a control, vibratome sections were incubated in Krebs–Ringer solution without BCH. The total DA content in the nervous tissue and in the incubation medium after an hour of incubation was considered an indicator of DA synthesis in vibratome sections of the striatum. The use of BCH in the incubation medium in this study led to a decrease in the total DA content in vibratome sections and in the incubation medium and, consequently, to a decrease in DA synthesis compared with the control. Further quantification showed an approximately 1.5-fold (32.9%) increase in the cooperative DA synthesis in the striatum of transgenic mice in the PD model compared with the control. Although this value was slightly lower than what we previously observed for the striatum of wild-type mice when modeling PD (51%) [23], we can conclude that, regardless of the mouse strain, the portion of the cooperative synthesis DA of the total synthesis of DA in the striatum increases after DAergic deafferentation.

## 4. Materials and Methods

### 4.1. Animals and Experimental Procedures

We used transgenic B6.B6D2-Tg(Th-EGFP)21-31Koba mice aged 20–24 weeks and weighing 25–30 g (RIKEN BRC, Tsukuba-shi, Ibaraki, Japan) (*n* = 58). In these animals, the GFP gene is under the promoter of the TH gene, which makes it possible to visualize neurons expressing the TH gene [40]. The transgenic mouse line was maintained by crossing B6.B6D2-Tg(Th-EGFP)21-31Koba mice with C57BL/6 mice (Laboratory Animal Farm Stolbovaya (SCBMT RAMS, Stolbovaya, Moscow Reg., Russia)). Transgenic animals were genotyped via amplification of tail tissue genomic DNA according to the RIKEN BRC protocol (protocol ID PS-05058).

**Figure 6:**
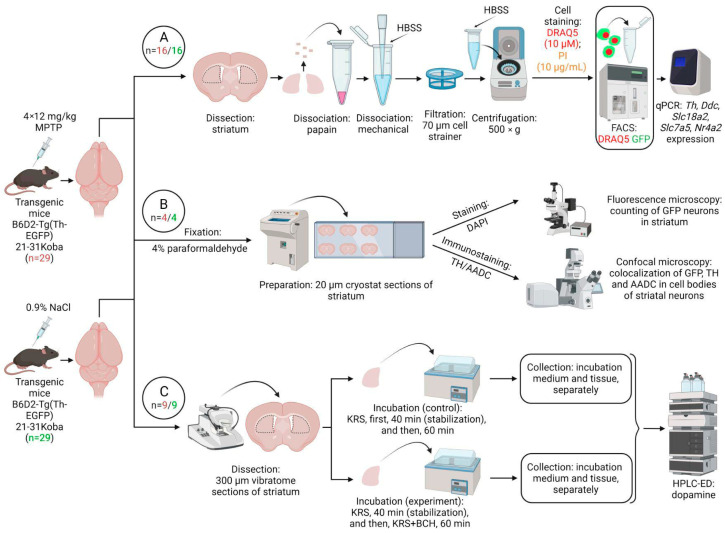
Design of experiments for transgenic mice expressing green fluorescent protein (GFP) gene under a promotor of the tyrosine hydroxylase (TH) gene. (**A**) Analysis of gene expression of proteins of dopaminergic phenotype in an isolated population of striatal neurons expressing the TH gene and stained with GFP (green) and nuclear dye DRAQ5 (red). (**B**) Morphological study of striatal neurons containing GFP, TH, and aromatic L-amino acid decarboxylase (AADC) with nuclei stained by DAPI. (**C**) Evaluation of cooperative dopamine synthesis using monoenzymatic striatal neurons synthesizing only TH or only AADC upon incubation of vibratome sections in Krebs–Ringer solution (KRS) with 0.5 mM 2-aminobicyclo[2.2.1]heptane-2-carboxylic acid (BCH), a competitive inhibitor of large amino acid transporter 1 (experiment), or without it (control). FACS, fluorescence-activated cell sorting; HBSS, Hanks’ Balanced Salt Solution; HPLC-ED, high-performance liquid chromatography with electrochemical detection; PI, propidium iodide; qPCR, quantitative polymerase chain reaction. *n*—number of animals in experimental (red)/or control (green) group.

The animals were randomly divided into an experimental group (n = 29) and a control group (*n* = 29). Mice of the experimental group were subcutaneously injected with the DAergic neurotoxin MPTP (Sigma-Aldrich, St. Louis, MO, USA) 4 times at a single dose of 12 mg/kg at 2 h intervals [25]. Mice of the control group were subcutaneously injected with 0.9% NaCl according to the same scheme. After the injections, mice of both groups were kept for 2 weeks under normal laboratory conditions (22 ± 1 °C, 12 h day/night cycle, free access to food and water) until biological material was obtained. The rostro-dorsal striatum (hereinafter, striatum) was used for sorting striatal neurons expressing the TH gene, followed by a quantitative polymerase chain reaction (qPCR). In addition, cryostat sections were prepared from the striatum of transgenic mice for subsequent immunocytochemical detection of DA synthesis enzymes. In addition, vibratome sections were prepared from the same material for subsequent flow incubation with a competitive inhibitor of LAT1 (Figure 6). To obtain the samples necessary for calibrating the sorter, we used the striatum obtained from C57BL/6 mice (*n* = 2) at the age of 20–24 weeks weighing 24–28 g.

### 4.2. Obtaining a Cell Suspension from the Striatum of Transgenic Mice

The dissociation of the striatum and the preparation of cell suspensions were carried out according to the protocol of Troshev et al. [21]. Briefly, transgenic mice of the experimental (*n* = 16) and control (*n* = 16) groups (Figure 6A) were decapitated under anesthesia with 2.4% isoflurane (Baxter, Deerfield, IL, USA). After that, the brain was removed, and the striatum was excised. One sample is a striatum isolated from both hemispheres obtained from two animals. The striatum was cut into small pieces with razor blades and incubated in 2 mg/mL papain solution (Sigma-Aldrich, St. Louis, MO, USA) for 30 min at 37 °C with constant stirring. The enzymatic action of papain was stopped by adding fetal bovine serum chilled to 4 °C (Gibco, Thermo Fisher Scientific, Waltham, MA, USA) in tubes at a final concentration of 10% (*vol.*/*vol.*). The resulting solution was stirred in a vortex. The tissue was precipitated and resuspended in 400 µL of chilled Hanks’ Balanced Salt Solution several times according to the previously described protocol. Then, mechanical dissociation of the tissue was carried out using mechanical pipette tips of different diameters. The resulting suspension was stirred and filtered through a moistened 70 μm cell strainer (Falcon, Corning, Corning, NY, USA). The suspension was centrifuged for 5 min at 500× *g* and 4 °C. The supernatant was removed, and the precipitate was resuspended in 100 µL Hanks’ Balanced Salt Solution containing 10 µM DRAQ5 (Abcam, Cambridge, UK). The suspension was incubated in this solution for 5 min at 37 °C with constant stirring. The cell suspension with DRAQ5-stained nuclei was stored in the cold in foil-wrapped tubes until cell sorting. Prior to cell sorting, the suspension volume was adjusted to 500 µL with chilled Hanks’ Balanced Salt Solution containing 10 µg/mL propidium iodide (PI, Sigma-Aldrich, St. Louis, MO, USA) as a marker of dead cells and incubated for 5 min at 4 °C. All solutions that were subsequently used to prepare samples for fluorescence-activated cell sorting (FACS), and qPCR contained 100 U/mL of the RNase inhibitor RiboLock (Thermo Fisher Scientific, Waltham, MA, USA).

### 4.3. Fluorescence-Activated Cell Sorting

Striatal neurons from transgenic mice containing GFP and stained with DRAQ5 but not stained with PI were sorted into tubes with a FACSAria III cell sorter (BD, Franklin Lakes, NJ, USA) using a 100 µm diameter nozzle at a pressure of 20–21 psi. Threshold staining levels for DRAQ5, GFP, and PI were determined by analyzing unstained cell suspension obtained from the striatum of C57BL/6 mice. For compensation, suspensions stained with only one of the dyes were used (compensation controls): DRAQ5, GFP, or PI (Figure S2). DRAQ5 was excited with a 640 nm laser and detected with a 670/30 nm band pass filter (DRAQ5 channel), while GFP was excited with a 488 nm laser and detected with a 530/30 nm band pass filter (GFP channel). PI was excited with a 488 nm laser and detected with a 585/42 nm band pass filter (phycoerythrin channel: PE). Histograms were constructed according to the distribution density of all detected events (all particles detected by the sorter), and the results were analyzed using the FlowJo v10 software (BD, Franklin Lakes, NJ, USA).

In total, 16 suspensions were prepared with sorted cells: each contained 2500 neurons stained with DRAQ5 containing GFP and unstained with PI (8 for the experimental group and 8 for the control group). All suspensions were sorted in TRI-reagent (Sigma-Aldrich, Saint Louis, MO, USA) and then vortexed, frozen in liquid nitrogen, and stored at −70 °C until RNA isolation.

### 4.4. Immunohistochemistry

Transgenic mice of the experimental (*n* = 4) and control (*n* = 4) groups were perfused under chloral hydrate anesthesia (Sigma-Aldrich, St. Louis, MO, USA) via the heart, first with 0.02 M phosphate buffer saline (PBS) (pH = 7.2–7.4) for 10 min and then with 4% paraformaldehyde in 0.1 M phosphate buffer (pH 7.2–7.4) for 10 min (Figure 6B). The animals were then decapitated; the brains were removed and post-fixed via immersion in the same fixative for 12 h at 4 °C, rinsed three times in PBS for 10 min each, immersed in 20% sucrose in PBS for 24 h at 4 °C, frozen in hexane at 40 °C, and maintained at 70 ° until immunocytochemistry.

Frontal cryostat sections, 20 µm thick, of the striatum were prepared on a cryostat (Leica CM1950, Leica Camera AG, Wetzlar, Germany) at a distance of 1.70 to 0.14 mm from the bregma in accordance with the mouse brain atlas [41]. Every fourth section, starting from the first section, was mounted on a glass slide and embedded in a medium containing 4′,6-diamidino-2-phenylindole (DAPI, Abcam, Cambridge, UK). Every fourth section, starting from the second section, was mounted on a glass slide for subsequent immunostaining for TH and AADC. For immunostaining, the sections were sequentially incubated in PBS containing (i) 1% sodium dodecyl sulfate (Sigma-Aldrich, St. Louis, MO, USA) for 5 min; (ii) 3% bovine serum albumin (BSA, Sigma-Aldrich, St. Louis, MO, USA) and 0.3% Triton X-100 (Sigma-Aldrich, St. Louis, MO, USA) for 1 h; (iii) sheep polyclonal anti-TH antibodies (1:1000, ab1542, Millipore, Burlington, MA, USA) and rabbit polyclonal anti-AADC antibodies (1:300, ab3905, Abcam, Cambridge, UK), 3% BSA, and 0.1% Triton X-100 for 20 h; and (iv) donkey anti-rabbit gamma globulin Alexa Fluor 555 antibodies (1:1000, A32794, Invitrogen, Thermo Fisher Scientific, Waltham, MA, USA) and donkey anti-sheep gamma globulin Alexa Fluor 633 antibodies (1:1000, A21100, Invitrogen, Thermo Fisher Scientific, Waltham, MA, USA) for 2 h. All incubations were carried out at 20 °C. After each incubation, except the last one, the sections were washed three times in PBS for a total of 45 min. After the last incubation, the sections were washed in PBS for one hour and placed in a medium containing DAPI.

### 4.5. Microscopy

#### 4.5.1. Fluorescence Microscopy of the Striatum of Transgenic Mice

Striatal neurons expressing the TH gene were examined under a fluorescent microscope (Leica DMi8 M, Leica Camera AG, Wetzlar, Germany) at a 20× objective magnification. Panoramic images were created using a 5 × 4 panel with a 20% overlap between photos. Panoramic images were created using the Stitching plugin and the FiJi software (available online: https://imagej.net/software/fiji/downloads, accessed on 7 October 2023).

#### 4.5.2. Counting the Bodies of Neurons Expressing the Tyrosine Hydroxylase Gene in the Striatum

The bodies of neurons expressing the TH gene were counted using the previously described method [21]. To sum up, neurons were counted in the striatum of animals of all groups on every fourth frontal section using the FiJi software. The images were preliminarily converted into an 8-bit format, and the fluorescence detection threshold was set so that the fluorescence intensity of neurons expressing the TH gene and containing GFP was higher than the fluorescence of unstained structures (autofluorescence). After that, all objects were counted that contained GFP, had an area of more than 10 μm^2^, and constituted the bodies of neurons.

#### 4.5.3. Confocal Microscopy of the Striatum of Transgenic Mice

To assess the colocalization of TH and AADC in GFP neurons, sections of the striatum obtained from mice of the experimental and control groups were examined and photographed under a confocal microscope (Zeiss LSM880, Zeiss, Oberkochen, Germany) using the following objectives: Plan-Apochromat 20×/0.8 M2 and Plan-Apochromat 63×/1.40 Oil DIC M27. Images were obtained separately in four channels. The signals from Alexa Fluor 633 were recorded in the first channel, the signal from Alexa Fluor 555 in the second channel, the signal from GFP in the third channel, and the signal from DAPI in the fourth channel. To study the topographic relationships between the labeled neurons (cell bodies), images of these neurons were studied under a confocal microscope on optical sections with a thickness of 2 μm at a 63× magnification with an additional 2× magnification. To amplify signal detection, a z-stack was made with a step of 1.4 μm, after which, the images of three stacks were stacked via the “orthogonal projection” method using the ZenBlue v 3.2.0.0000 software (Zeiss, Oberkochen, Germany). Further image processing was performed using the same software.

### 4.6. Flow Incubation of Striatum Sections

In transgenic mice of the experimental and control groups (*n* = 9 for each group) (Figure 6C), four 300 μm thick frontal sections were obtained from each brain at the level of the striatum using a vibratome (Leica VT1200S, Leica Biosystems, Wetzlar, Germany). The sections were prepared in Krebs–Ringer solution cooled to 4 °C: NaCl, 120 mM; KCl, 4.8 mM; CaCl_2_, 2 mM; MgSO_4_, 1.3 mM; NaHCO_3_, 25 mM; D-glucose, 10 mM; HEPES, 20 mM; and ascorbic acid, 0.1 mM (all from Sigma-Aldrich, St. Louis, MO, USA) at pH = 7.3. The striatum was excised from these sections in a cooled Krebs–Ringer solution under the control of a Leica M60 stereomicroscope.

Four sections of the striatum from one hemisphere of the brain were included in each sample. Sections of the striatum obtained from the left hemisphere were used in the control, and sections from the right hemisphere of the brain were used in the experiment. In both cases, the sections were incubated in thermostated chambers connected to a multichannel pump (Ismatec, Wertheim, Germany) at 37 °C and at an incubation medium flow rate of 100 µL/min. The sections were incubated for the first 40 min in Krebs–Ringer buffer for functional stabilization and for the next 60 min in Krebs–Ringer buffer containing 0.04% NH_4_OH (control) or in Krebs–Ringer buffer containing 0.04% NH_4_OH and 0.5 mM BCH (Sigma-Aldrich, St. Louis, MO, USA) LAT1 inhibitor (experiment) (Figure 1C). NH_4_OH was used to dissolve BCH [42]. After the end of the incubation, 3,4-dihydroxybenzylamine hydrobromide (Sigma-Aldrich, St. Louis, MO, USA) at a final concentration of 10 nM was added as an internal standard to the incubation medium collected over 60 min. After the incubation, the resulting solution and sections were frozen in liquid nitrogen. The incubation medium and the sections were stored at −70 °C until HPLC with electrochemical detection was performed.

### 4.7. High-Performance Liquid Chromatography with Electrochemical Detection

High-performance liquid chromatography with electrochemical detection was used to determine DA in the incubation medium and in the striatum sections of transgenic mice after incubation with and without BCH. Samples of the incubation medium were prepared via solid-phase extraction using aluminum oxide according to the protocol described earlier [23]. To measure the DA content in the striatal sections, frozen tissue was homogenized using an ultrasonic homogenizer (Hielscher UP100H, Hielscher Ultrasonics GmbH, Teltow, Germany) at 4 °C in 400 μL of a 0.1 M HClO_4_ solution containing 250 pmol/mL of 3,4-dihydroxybenzylamine hydrobromide. In total, 30 μL of the homogenate was then taken to measure the concentration of total protein using a solution of bicinchoninic acid (BCA method) [43]. The remainder of the homogenate was centrifuged for 20 min at 18,000× *g* and 4 °C, and the supernatant was collected for further analysis.

Determination of DA content was carried out on a reverse phase column (100 × 4 mm ReproSil-Pur C18, 3 μm) (Dr. Maisch, Ammerbuch, Germany). The mobile phase was 0.1 M citrate–phosphate buffer containing 0.25 mM sodium salt of 1-octane sulfonic acid, 0.1 M ethylenediaminetetraacetic acid, and 9% acetonitrile (pH = 2.55, all reagents from Sigma-Aldrich, St. Louis, MO, USA). A Decade II electrochemical detector (Antec Leyden, Leyden, The Netherlands) was equipped with a working glassy carbon electrode (+0.85 V) and an Ag/AgCl reference electrode. The peaks of the catecholamines, their metabolites, and the internal standard were identified by the time of their release into the solution. The DA content was calculated from the ratio of the peak areas in the sample to the standards. The peak areas were measured using the LabSolutions v 5.87 software (Shimadzu, Kyoto, Japan). The DA content was normalized to the concentration of total protein for the striatum sections or to the volume of the incubation medium and the concentration of total protein for the incubation medium.

The level of DA synthesis caused by non-dopaminergic striatal neurons was calculated as follows:

ΔDA = (C_DA Tbch-_ + C_DA IMbch-_) − (C_DA Tbch+_ + C_DA IMbch+_),

where C_DA Tbch-_ is the DA content in the striatum sections (tissue) after incubation without BCH;

C_DA IMbch-_ is the DA content in the incubation medium after incubation without BCH;
C_DA Tbch+_ is the DA content in the striatum sections (tissue) after incubation with the addition of BCH;
C_DA IMbch+_ is the DA content in the incubation medium after incubation with the addition of BCH.

The contribution of DA synthesis by monoenzymatic neurons to the total DA synthesis in the striatum was calculated as follows:

K_DA_ = ((C_DA Tbch-_ + C_DA IMbch-_) − (C_DA Tbch+_ + C_DA IMbch+_))/(C_DA Tbch-_ + C_DA IMbch-_)

### 4.8. RNA Isolation and Quantitative Polymerase Chain Reaction

The volume of thawed TRI-reagent containing sorted striatal neurons was adjusted to 1 mL using TRI-reagent. In total, 100 μL of 1-bromo-3-chloropropane (Sigma-Aldrich, St. Louis, MO, USA) was then added, and the mixture was vortexed and incubated for 15 min at 20 °C. After that, the phases were separated by centrifuging the resulting solution for 15 min at 21,000× *g* and 4 °C. The aqueous phase containing RNA was transferred to a new tube, and 500 µL of isopropyl alcohol (Sigma-Aldrich, St. Louis, MO, USA) and 1 µL of glycogen (Thermo Fisher Scientific, Waltham, MA, USA) were added for better RNA precipitation. The solution was stirred and incubated for 10 min at 20 °C, after which, the RNA was precipitated for 10 min at 21,000× *g* and 4 °C. The supernatant was collected, and the precipitate was washed three times with 1 mL of 80% ethanol and centrifuged for 10 min at 21,000× *g* and 4 °C. After the last centrifugation, the ethanol was removed, and the RNA precipitate was air-dried for 15 min. The RNA concentration in all samples was measured using a NanoDrop 8000 spectrophotometer (Thermo Fisher Scientific, Waltham, MA, USA). The rest of the genomic DNA was removed with DNase I RNase-free (Thermo Fisher Scientific, Waltham, MA, USA) according to the manufacturer’s recommendations. Complementary DNA was synthesized from 150 ng of total RNA using the Maxima H Minus First Strand cDNA synthesis kit (Thermo Fisher Scientific, Waltham, MA, USA) according to the manufacturer’s recommendations. Complementary DNA concentration was measured using a NanoDrop 8000 spectrophotometer. qPCR was performed with a QuantStudio 12k Flex amplifier (Applied Biosystems, Waltham, MA, USA), QuantStudio 12k Flex (Applied Biosystems, Waltham, MA, USA) using the qPCRmix-HS SYBR + LowROX reaction mixture (Evrogen, Moscow, Russia) and oligonucleotide primers (Evrogen, Moscow, Russia) (Table 1). In total, 500 ng of cDNA was taken into the reaction.

As a housekeeping gene, we used the cytochrome C1 gene, the expression of which is least susceptible to changes during neurodegeneration in comparison with other genes [44]. In the sorted TH-expressing striatal neurons of mice in the experimental group and the control group, the gene expression levels of the following proteins were evaluated: TH, VMAT2, AADC, LAT1, and transcription factor Nurr1. Gene expression levels are expressed as 2^–ΔΔCt^ values normalized to the expression of *Cyc1* as a housekeeping gene. Formulas (1) and (2) were used for calculating ΔΔCt as follows:

∆Ct = (Ct(*gene*) − Ct(*Cyc1*) (1)

∆∆Ct = (∆Ct(sample) − ∆Ct(medium control))(2)

The results were calculated as the geometric mean of the group [45] and are presented as fold changes with respect to the control.

### 4.9. Statistical Analysis

Statistical analysis was performed using the GraphPad Prism 6 software (GraphPad Software, San Diego, CA, USA). The groups were compared for normality by using the Shapiro–Wilk test. For pairwise comparison, we used the paired *t*-test. For multiple comparisons, we used the Friedman test with Dunn’s multiple comparisons test. The results are presented as mean ± SEM or as mean with interquartile range. Differences were considered significant at *p* ≤ 0.05.

## 5. Conclusions

The purpose of this work was to study compensatory processes in the striatum of mice aimed at maintaining DA homeostasis in this center for the regulation of motor function in a model of PD. The level of DA in the striatum of mice in the PD model decreased by more than 80%, as in patients. The main focus of our work was on studying the functional activity of striatal non-DAergic neurons by assessing the gene expression and protein synthesis of the DAergic phenotype. Significant progress in these studies was achieved because of the use of transgenic mice expressing the GFP gene under the TH gene promoter. We showed that, when modeling PD in transgenic mice, the number of neurons expressing the TH gene increases 1.9 times, which, apparently, is a compensatory reaction in response to the DAergic denervation of the striatum. The greatest increase in the number of these neurons, almost four times, was observed in the most rostral segment of the dorsal striatum, the most denervated in PD. These data suggest that DAergic afferents inhibit TH expression in striatal neurons. An increase in the functional activity of striatal neurons expressing the TH gene in PD modeling is also evidenced by a 2.5-fold increase in the expression of the TH and Nurr1 genes and a 1.45-fold increase in the expression of the LAT1 gene. From a physiological point of view, it is important to note that, immunohistochemically, we were able to detect this in the cell bodies of neurons and not only in their processes, as in the control: (i) TH in GFP-containing neurons, (ii) TH and AADC in neurons containing GFP, and (iii) AADC in neurons without GFP. In addition, following the DAergic deafferentation of the striatum, we showed that there is a compensatory increase in cooperative DA synthesis caused by striatal neurons expressing only TH and neurons expressing AADC. This work opens up broad prospects for the further study of compensatory processes in the striatum that maintain DA homeostasis in PD. In addition, fundamental knowledge about the mechanisms of the neuroplasticity of the striatum during its DAergic denervation in PD can be used to improve the treatment of this severe, socially significant disease.

## Figures and Tables

**Table 1:** Oligonucleotide primers used for setting qPCR.

Gene Designation According to the NCBI Database	Protein	Forward Primer	Reverse Primer
*Cyc1*	Cytochrome C1	5′-GCGGCCAGGGAAGTTGT-3′	5′-GCCAGTGAGCAGGGAAAATAC-3′
*Th*	Tyrosine hydroxylase	5′-TCAGAGGAGCCCGAGGTC-3′	5′-GGGCGCTGGATACGAGAG-3′
*Ddc*	Aromatic L-amino acid decarboxylase	5′-TCCCCACGGCTAGCTCATACCC-3′	5′-TTCCCCAGCCAGTCCATCATCA-3′
*Nr4a2*	Transcription factor Nurr1	5′-CCGAAGAGCCCACAGGAT-3′	5′-CCATAGAGCCGGTCAGGAG-3′
*Slc18a2*	Vesicular monoamine transporter 2	5′-ATTGGCTTTCCTTGGCTCAT-3′	5′-GGTACGGCTGGACATTATTCTG-3′
*Slc7a5*	Large amino acid transporter 1	5′-CTCCCGGTGTTCTTTATCCTG-3′	5′-AGAATCCACTTGGGCTTGTT-3′

## Data Availability

The data presented in this study are available upon request from the corresponding author. The data are not publicly available because of legal issues.

## Supplementary Materials

The following supporting information can be downloaded at https://www.mdpi.com/article/10.3390/ijms242216245/s1.

## Abbreviations

AADC	aromatic L-amino acid decarboxylase
BCH	2-aminobicyclo[2.2.1]heptane-2-carboxylic acid
DA	dopamine
GFP	green fluorescent protein
LAT1	large amino acid transporter 1
L-DOPA	L-3,4-dihydroxyphenylalanine
MPTP	1-methyl-4-phenyl-1,2,3,6-tetrahydropyridine
PI	propidium iodide
qPCR	quantitative polymerase chain reaction
TH	tyrosine hydroxylase
VMAT2	vesicular monoamine transporter 2
