# Supplementary material for: Compensatory Processes in Striatal Neurons Expressing the Tyrosine Hydroxylase Gene in Transgenic Mice in a Model of Parkinson’s Disease
Source: Int J Mol Sci. 2023 Nov 13;24(22):16245. doi: 10.3390/ijms242216245 (PMC10671746; doi:10.3390/ijms242216245)
Supplement: Supplementary file 1 [file ijms-24-16245-s001.zip › Supplementary Figures.pdf]

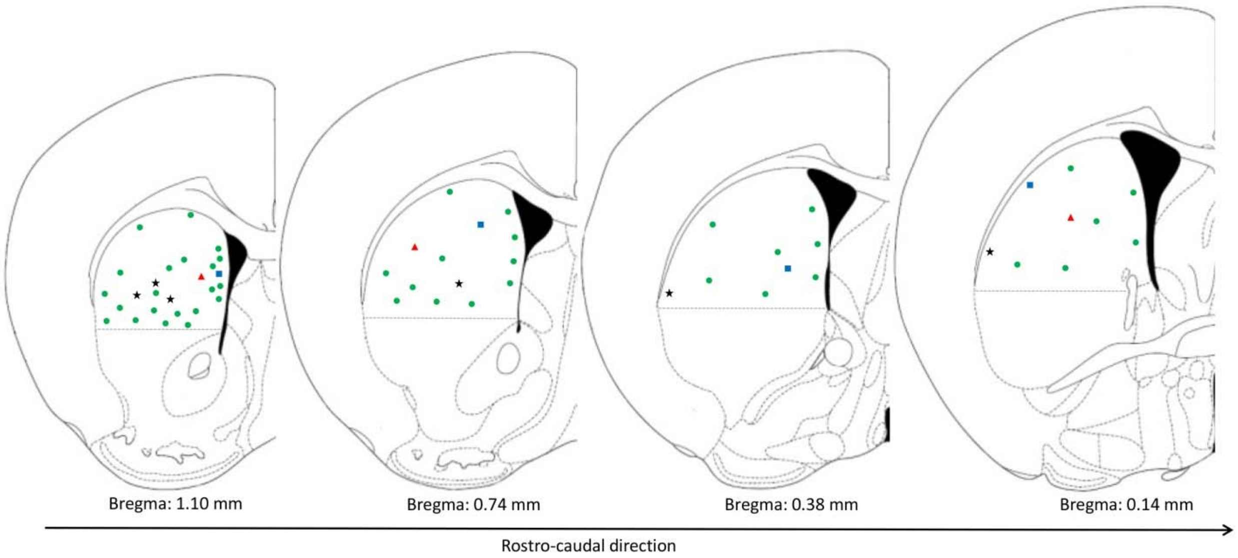

Figure S1. Schematic representation of the distribution in the rostro-caudal direction of the dorsal striatum of transgenic mice of neurons containing: GFP, TH-immunopositive and AADC-immunopositive materials (black asterisks); GFP and TH-immunopositive material but no AADC-immunopositive material (red triangles); GFP but without TH-immunopositive and AADC-immunopositive materials (green circles); TH-immunopositive material but without GFP and AADC-immunopositive material (blue squares). n = 1.

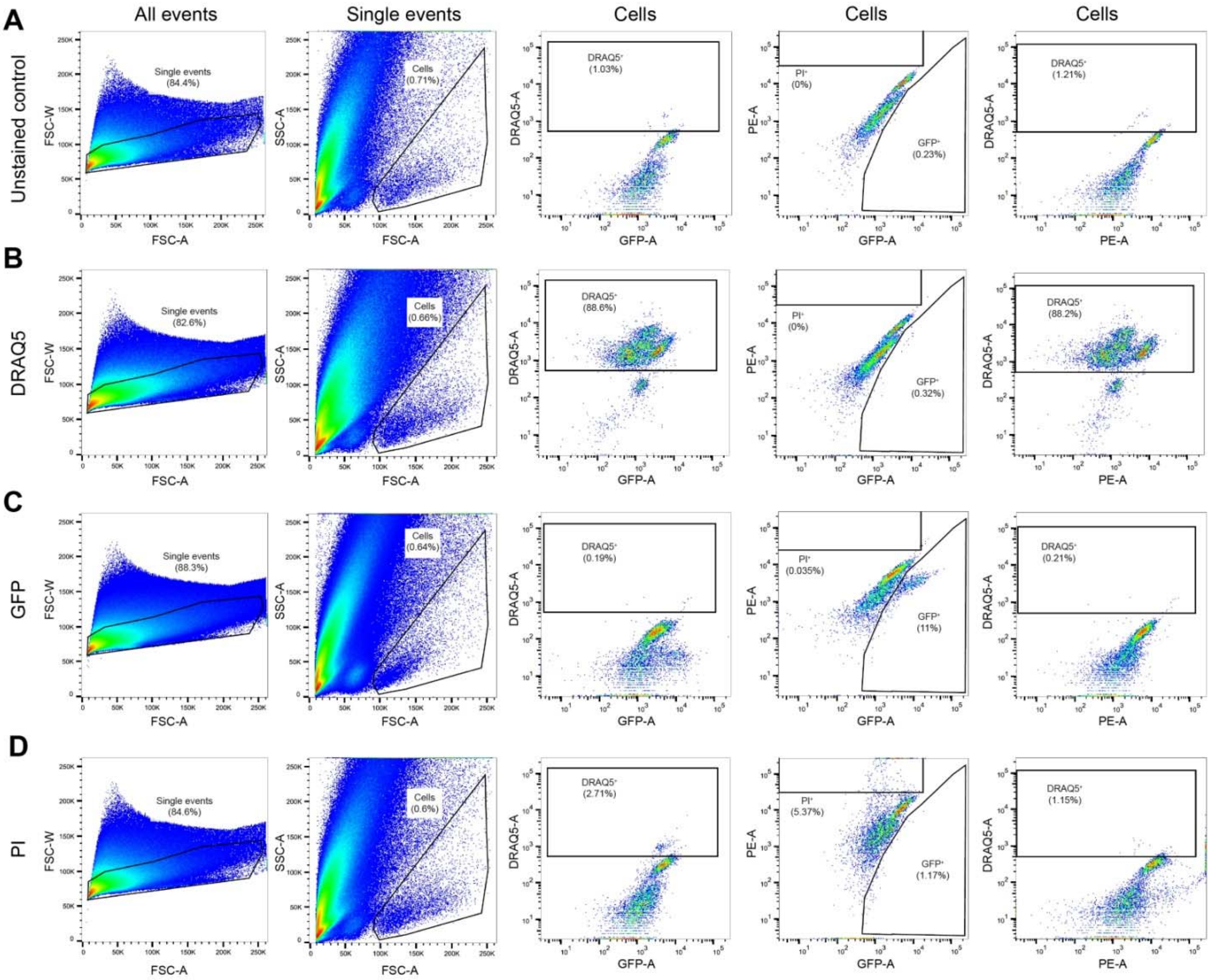

Figure S2. Unstained control and compensation controls for each fluorophore. (**A**) Unstained control. (**B**) Staining of cells with DRAQ5. (**C**) Staining of cells with GFP. (**D**) Staining of cells with PI. FSC, forward scatter; PE, phycoerythrin (detection channel); SSC, side scatter.
